# Supplementary material for: Crank Nicholson scheme to examine the fractional-order unsteady nanofluid flow of free convection of viscous fluids
Source: PLoS One. 2022 Mar 1;17(3):e0261860. doi: 10.1371/journal.pone.0261860 (PMC8887771; doi:10.1371/journal.pone.0261860)
Supplement: S1 Abbreviations — (DOCX) [file pone.0261860.s001.docx]

**Abbrevations**

| **Name** | **Symbol** | **Unit** | **Name** | **Symbols** |
| --- | --- | --- | --- | --- |
| Distance | $d$ | m | Fractional differential equation | FDE |
| Constant temperature | $T_{0}$ | $K/C$ | Laplace transformation method | LTM |
| Constant concentration | $C_{0}$ | $mol/m^{3}$ | Crank Nicolson method | CNM |
| Temperature | $T$ | $K/C$ | Nano fluid | nf |
| Concentration | $C$ | $mol/m^{3}$ | fluid | f |
| Velocity | $u$ | $m/s$ | Solid particle | s |
| Gravitational Acceleration | $g$ | $m/s^{2}$ | Heat transfer constant | $\beta$ |
| Kinematic viscosity | $\nu$ | $m^{2}/s$ | Rate of chemical reaction | $\gamma$ |
| Density | $\rho$ | $kg/m^{3}$ | Order of chemical reaction | $n$ |
| Electrical conductivity | $\sigma$ | $S/m$ | Porosity | $\phi_{m}$ |
| Heat capacity | $C_{p}$ | $J/K$ | Mass diffusion | $D$ |
| Thermal conductivity | $k$ | $W/mK$ | Absorption coefficient | $k_{\lambda}$ |
| Current density | $\boldsymbol{J}$ | $A/m^{2}$ | Plank function | $e_{b\lambda}$ |
| Viscosity | $\mu$ | $PI$ | fractional parameter | $\alpha$ |
| Magnetic field | $\boldsymbol{B}$ | tesla(T) | volume fraction | $\phi$ |
| Electric field | $E$ | $N/C$ | copper | $Cu$ |
| Lewis number | $Le$ |  | silver | $Ag$ |
