# Supplementary material for: Crank Nicholson scheme to examine the fractional-order unsteady nanofluid flow of free convection of viscous fluids
Source: PLoS One. 2022 Mar 1;17(3):e0261860. doi: 10.1371/journal.pone.0261860 (PMC8887771; doi:10.1371/journal.pone.0261860)
Supplement: Attachment — Submitted filename: Response Letter.docx [file pone.0261860.s002.docx]

**Response to reviewer’s comments**

**Manuscript ID:** PONE-D-21-31025

**Journal Name:** PLOS ONE

**Dear Editor-in-Chief,**

Thank you for your useful comments on our manuscript. We have considered your additional editorial comments and made the following changes in our paper entitled “Crank Nicholson Scheme to Examine the Fractional-order Unsteady Nanofluid Flow of Free Convection of Viscous Fluids". We have modified the manuscript accordingly, and detailed corrections are listed below point by point. All the changes of are highlighted in red colour.

**Additional Editor Comments & Response:**

1. Strengthen the mathematical modelling of the problem.

**Response:** Geometry of the problem has been added to fix the query.

2. Update the introduction with relevant references only. Also arrange the references in proper format.

**Response:** Introduction section has been updated according to the instructions. The format of the references has been improved.

3. Validate the results.

**Response:** Graphical results and further analysis on the base of presented figures has been added.

**With Regards,**

Tamour Zubair
